# Unilateral cross bite treated by corticotomy-assisted expansion: two case reports

**DOI:** 10.1186/1746-160X-6-6

**Published:** 2010-05-19

**Authors:** Ali H Hassan, Ali T AlGhamdi, Ahmad A Al-Fraidi, Aziza Al-Hubail, Manar K Hajrassy

**Affiliations:** 1 Preventive Dental Sciences Department, Faculty of Dentistry, King Abdulaziz University, Jeddah, Saudi Arabia; 2 Oral Basic and Clinical Sciences Department, Faculty of Dentistry, King Abdulaziz University, Jeddah, Saudi Arabia; 3 Saudi Board in Orthodontics, Faculty of Dentistry, King Abdulaziz University, Jeddah, Saudi Arabia

## Abstract

**Background:**

True unilateral posterior crossbite in adults is a challenging malocclusion to treat. Conventional expansion methods are expected to have some shortcomings. The aim of this paper is to introduce a new technique for treating unilateral posterior crossbite in adults, namely, corticotomy-assisted expansion (CAE) applied on two adult patients: one with a true unilateral crossbite and the other with an asymmetrical bilateral crossbite, both treated via modified corticotomy techniques and fixed orthodontic appliances.

**Methods:**

Two cases with asymmetric maxillary constriction were treated using CAE.

**Results:**

In both cases, effective asymmetrical expansion was achieved using CAE, and functional occlusion was established as well.

**Conclusions:**

Unilateral CAE presents an effective and reliable technique to treat true unilateral crossbite.

## Background

Unilateral posterior crossbite is not an uncommon malocclusion encountered in daily orthodontic practice. Several studies reported a prevalence that varied between 8% and 23% [1-4]. The condition may result from dental tipping, a skeletal deficiency, or a cleft palate. A unilateral posterior crossbite involving multiple teeth can be classified as either a functional posterior crossbite or a true unilateral crossbite [5]. In a functional posterior crossbite, the presence of an occlusal interference causes a shift of the mandible upon closure [5-7]. Bilateral maxillary expansion is usually recommended as the standard treatment for a functional crossbite since the discrepancy between the maxillary width and the mandibular width is usually due to insufficient maxillary width [6-13]. A true unilateral crossbite is more problematic and requires unilateral expansion, which cannot be achieved using the conventional expansion appliances [14-16]. Over-correction on the unaffected side [14-16] is a common consequence that may lengthen the overall treatment time and is difficult to correct. Several modifications of expansion appliances were performed in attempts to produce differential unilateral effects. These include the use of a removable appliance with unilateral finger springs [5,6] , a removable plate sectioned asymmetrically with jack-screw [5,6] , unilateral cross elastics and a quad-helix appliance with different arm lengths [5,6]. Unfortunately, these methods are not adequate due to several factors such as patient compliance and occurrence of undesirable tooth movement.

The development of corticotomy-assisted orthodontics has provided new solutions to many limitations in the orthodontic treatment of adults. The conventional non-surgical method of slow expansion used in adults is a problematic, limited and inefficient method, which takes a long time and might compromise periodontal health if done beyond a few millimeters [17]. Corticotomy-assisted expansion is an optimal way to treat mild to moderate maxillary transverse deficiency in adults with greater stability and without compromising periodontal health. Although corticotomy is an old technique dating back to the early 1900s, it was not properly introduced until Wilcko developed the patented technique named Accelerated Osteogenic Orthodontics (AOO) [18] , also called Periodontally Accelerated Osteogenic Orthodontics (PAOO)™ [19]. This technique was originally designed to enhance tooth movement, subsequently reducing treatment time via inducing cortical bone injury through linear cutting (corticotomy) and then performing orthodontic treatment. Frost [20] found a direct correlation between the severity of bone injury and the intensity of its healing response, which occurred mainly as a reorganized activity and accelerated bone turnover at the surgical site. This type of healing response was named "Regional Acceleratory Phenomenon" (RAP) and was defined as a temporary phenomenon of increased localized remodeling to rebuild the surgical site [21]. Wilcko [18,19] noticed that the reduced mineralization created by the corticotomies (osteopenia) of the alveolar bone housing the involved teeth and the subsequent RAP were the reasons behind the rapid tooth movement following corticotomies.

Evidence of the success of corticotomy as an aid to orthodontic treatment is not well documented. Few published clinical reports are available in the literature [18-20,22-25].

Decreased cortical resistance, increased bone remodeling, and bone augmentation seem to allow safer and stable expansion in skeletally mature patients where slow palatal expansion is ineffective, dangerous, and unstable. In addition, corticotomy can be a good choice of treatment to provide differential expansion as well as unilateral expansion in a more controlled way than conventional expansion since tooth movement is expected to be enhanced more at the corticomized site than at the non-corticomized site. Based on this proposed idea and the concept of PAOO, we suggest a technique named corticotomy-assisted expansion (CAE) as an effective treatment modality for unilateral crossbite in adults.

The aim of this paper is to introduce a new technique for treating unilateral posterior crossbite in adults, namely, corticotomy-assisted expansion (CAE) applied on two adult patients: one with a true unilateral crossbite and the other with an asymmetrical bilateral crossbite, both treated via modified corticotomy techniques and fixed orthodontic appliances.

### Case 1

#### History and Diagnosis

A 21-year-old female patient visited the orthodontic clinic with a chief complaint of frequently biting her cheek. Her medical and dental history was insignificant except for the extraction of the upper and lower left first molars and the upper right third molar. The anterior teeth were restored by ceramic veneers, which were not satisfactory. She had undergone multiple root canal treatments. Temporomandibular joints were healthy except for TMJ clicking. The patient was periodontally healthy

**Figure 1:**
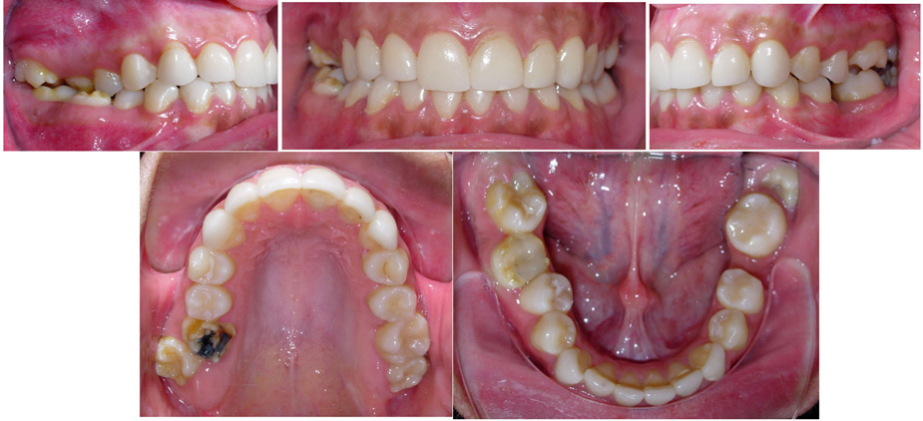
**Initial intra-oral composite photograph of case 1**.

Extra-oral examination revealed good facial proportions with a slightly convex profile and an acute nasolabial angle. The upper midline was centered to the facial midline, and the lower midline was deviated 2 mm to the right of the upper midline. (Figure 1)

**Figure 2:**
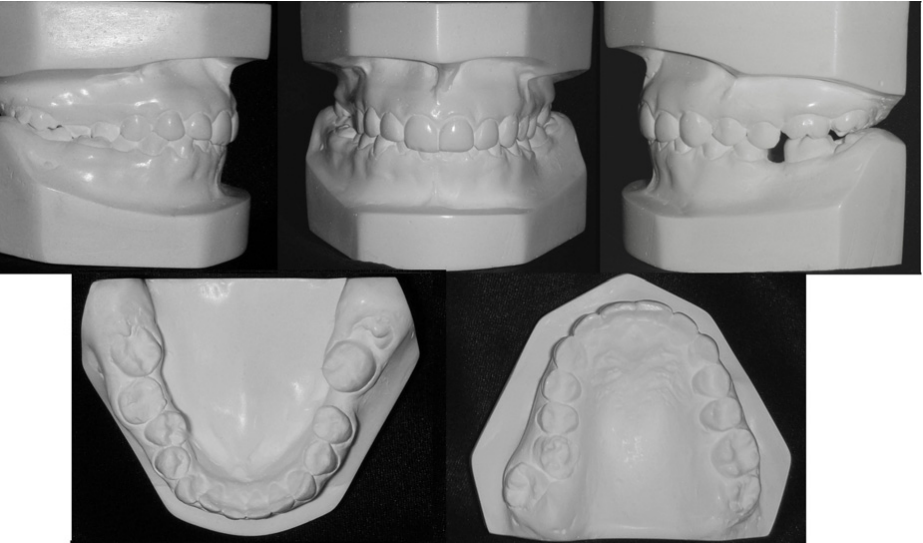
**Initial study model of case 1**.

**Figure 3:**
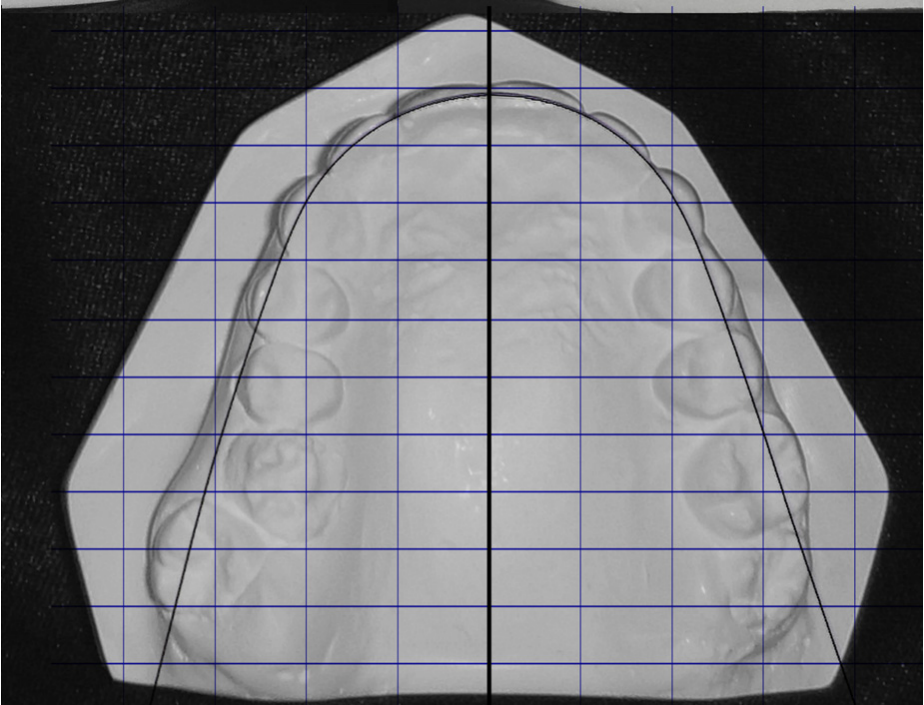
**The unilateral asymmetry of the upper arch is shown**.

**Figure 4:**
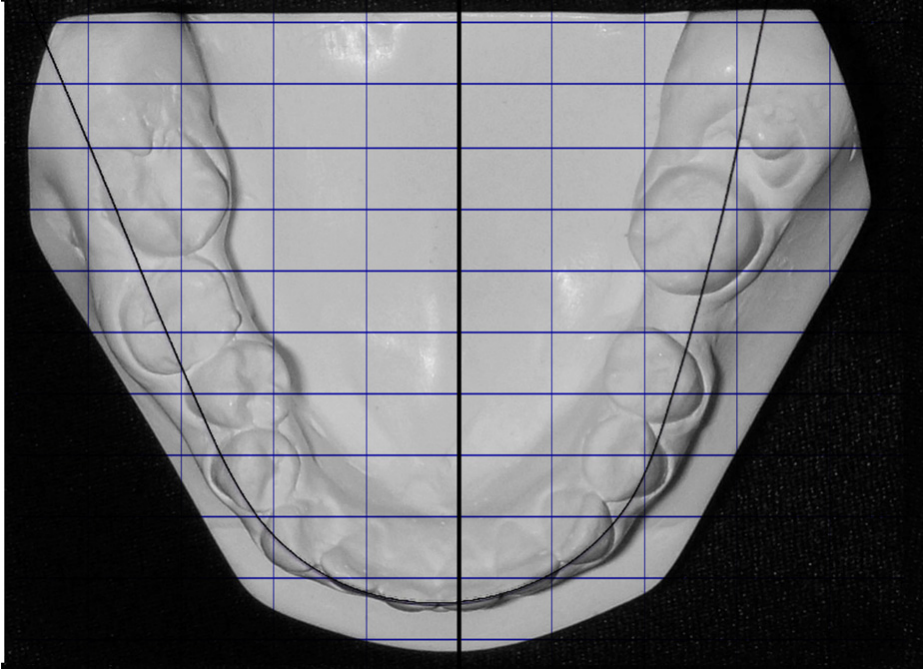
**The lingually positioned lower right 2^nd ^premolar and the rotation of the right 2^nd ^molar, masked the severity of the cross-bite**.

Dentally, the patient had Class I canines and buccal segment relationships, a 4-mm deep bite, a normal overjet, and a normal curve of Spee. There was 2 mm of crowding in the lower anterior segment, while the remaining space for the missing tooth number 37 was about 4 mm. There was a unilateral posterior crossbite on the right side due to unilateral constriction of the maxillary arch, compensated for by lingual tipping of the lower right first and second premolars and lower right second molar (Figure 2, 3, 4).

**Figure 5:**
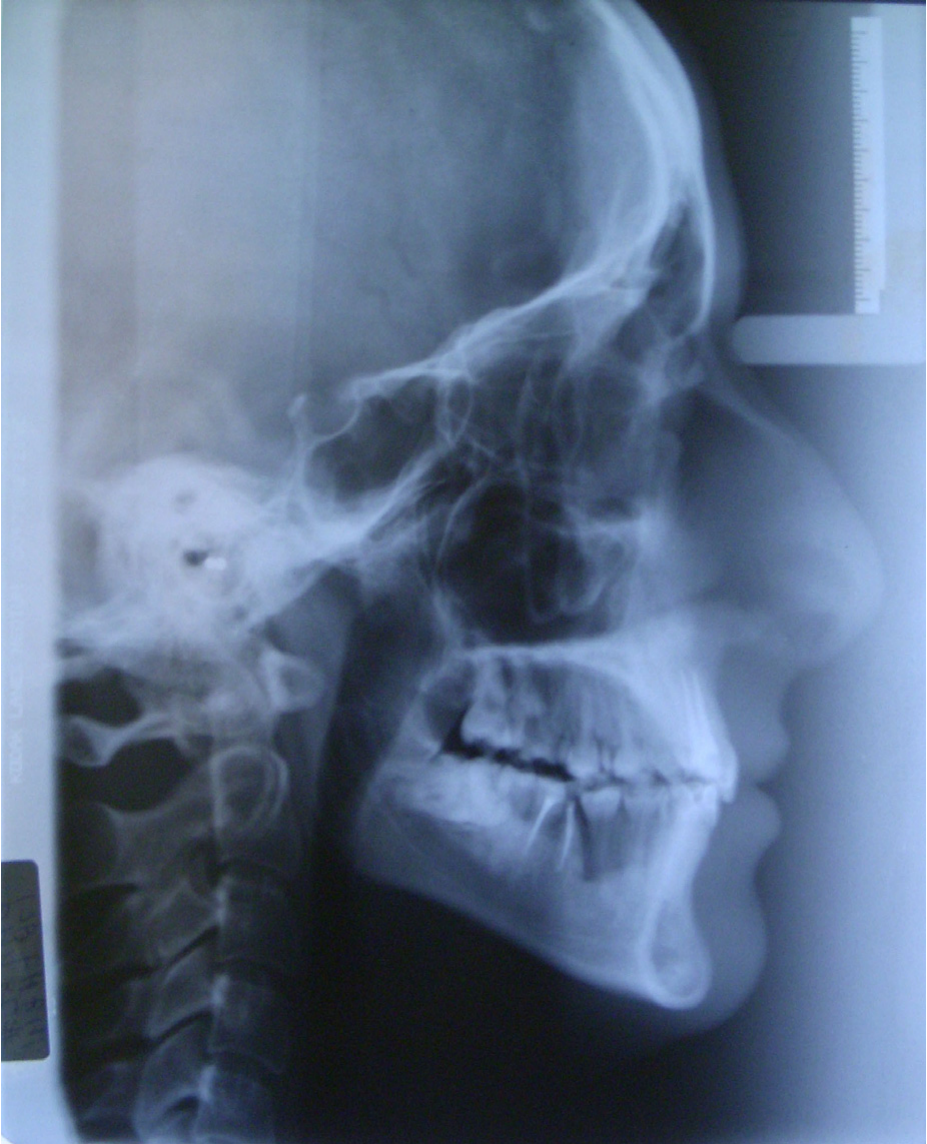
**Initial cephalogram of case 1**.

**Figure 6:**
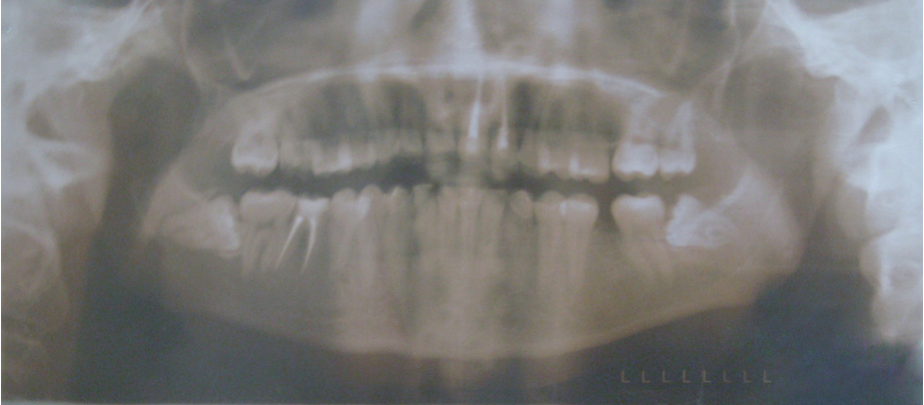
**Initial OPG of case1**.

Radiographically, the patient had a Class I skeletal relationship with normal mandibular plane and lower facial height. Upper and lower incisors were retroclined and retruded (Table 1). The lower left third molar was partially impacted and in a mesioangular direction. The lower right third molar was mesioangularly impacted (Figure 5, 6).

**Table 1:** Initial and final cephalometric readings of case 1.

Measurements	Norms*	Pre	Post
**Facial angle NPog/SN**	80°°	80°	80°

**SNA**	82°	81°	80°

**SNB**	80°	79°	78°

**ANB**	2°	2°	2°

**NA/APog**	0°	5°	4°

**Mandibular Plane to FH**	25°	30°	31°

**Mandibular Plane to SN**	32°	38°	37°

**Y Axis (SGn/SN)**	60° - 66°	67°	67°

**U Incisor to SN**	103°	112°	108°

**U Incisor To NA angle**	22°	28°	25°

**U Incisor to NA Distance**	4 mm	6 mm	5 mm

**L Incisor to Mandibular Plane**	90°	95°	90°

**L incisor to NB Angle**	25°	34°	28°

**L incisor to NB Distance**	4 mm	12 mm	7 mm

**Inter-incisal Angle**	130-132°	116°	125

**ANS to Gn/N to Gn**	57%	57%	57%

* Steiner CC, Cephalometric for you and me. American journal of orthodontics. 1960; 46: 721

#### Treatment Objectives

- To correct posterior crossbite via unilateral CAE.

- To resolve crowding, eliminate rotations, and correct lower midline deviation.

- To close the residual spaces of old extraction sites.

- To correct the deep bite.

- To upright the lower left third molar and extract the contralateral one.

- To achieve functional occlusion with maximum intercuspation, minimal overbite, and minimal overjet.

#### Treatment Plan and Progress

**Figure 7:**
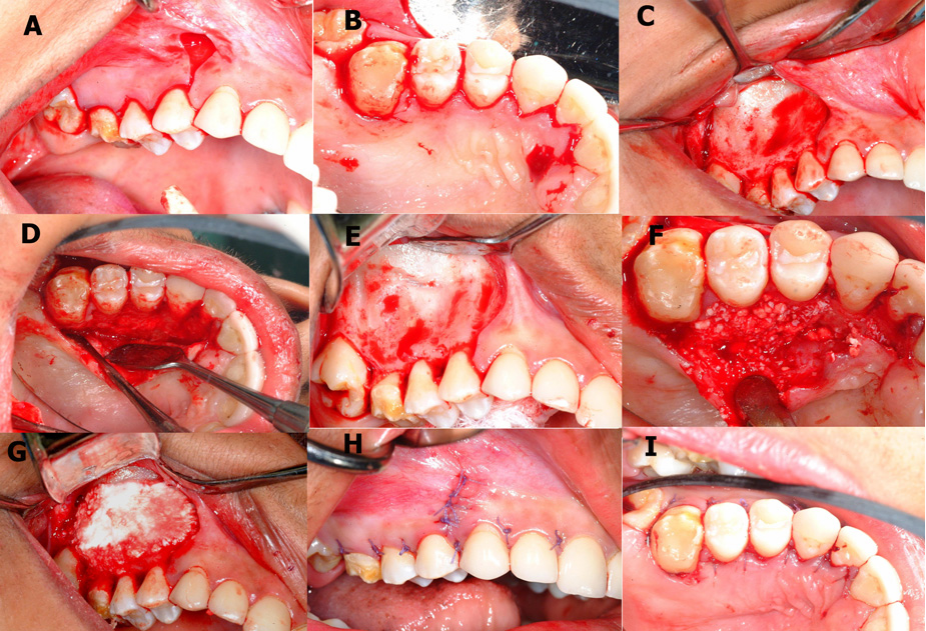
**Surgical procedure of CAE.** A &B: buccal and palatal incisions are made. C & D: full thickness flap is reflected. E: selective alveolar decortications lines and points are made. F & G: bone graft is placed. H & I: flap is sutured back.

**Figure 8:**
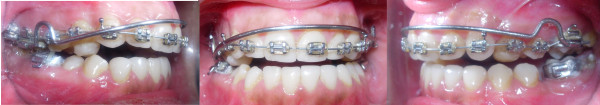
**Heavy labial bow used as the expanding appliance for case 1**.

**Figure 9:**
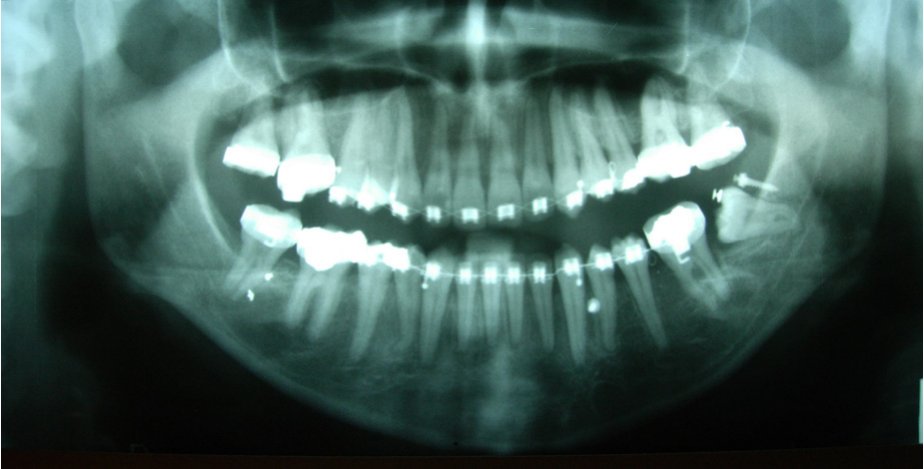
**OPG showing the use of a miniscrew to upright the lower left third molar**.

Corticotomy was performed on the buccal and palatal side of the right maxillary segment as described by Wilcko [18] (Figure 7). Expansion started 10 days after corticotomy and was performed using fixed orthodontic appliance (Victory Series™ low profile brackets, 3 M Unitik, St. Paul, Minnesota, USA 0.018 × 0.025-in brackets) and a heavy labial arch wire (0.040-in Stainless Steel wire) (Figure 8). Cross bite correction was achieved in 10 weeks. The lower left third molar was uprighted using a miniscrew, that was 1.6 mm in diameter and 8 mm in length (RMO^®^, Denver, USA), and an open coil spring. The lower right third molar was extracted (Figure 9). Leveling, aligning, arch coordination, and finishing were continued using the fixed orthodontic appliance and intermaxillary elastics. For retention, an upper wrap-around retainer and a lower fixed retainer from canine to canine were used.

#### Results

**Figure 10:**
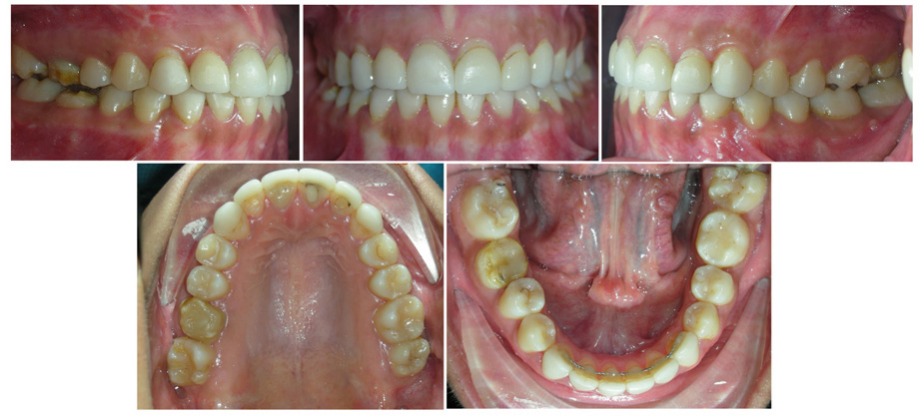
**Final intraoral composite photograph of case 1**.

**Figure 11:**
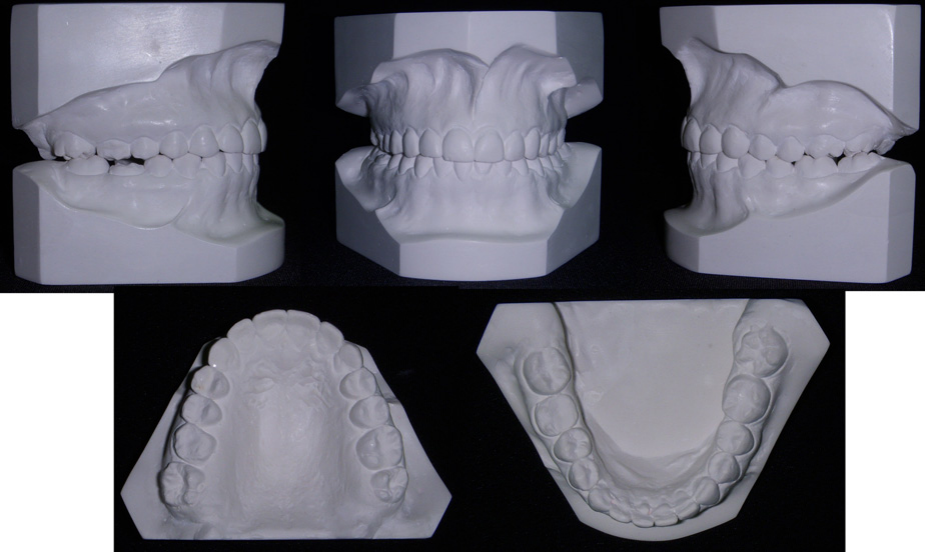
**Final study model of case 1**.

**Figure 12:**
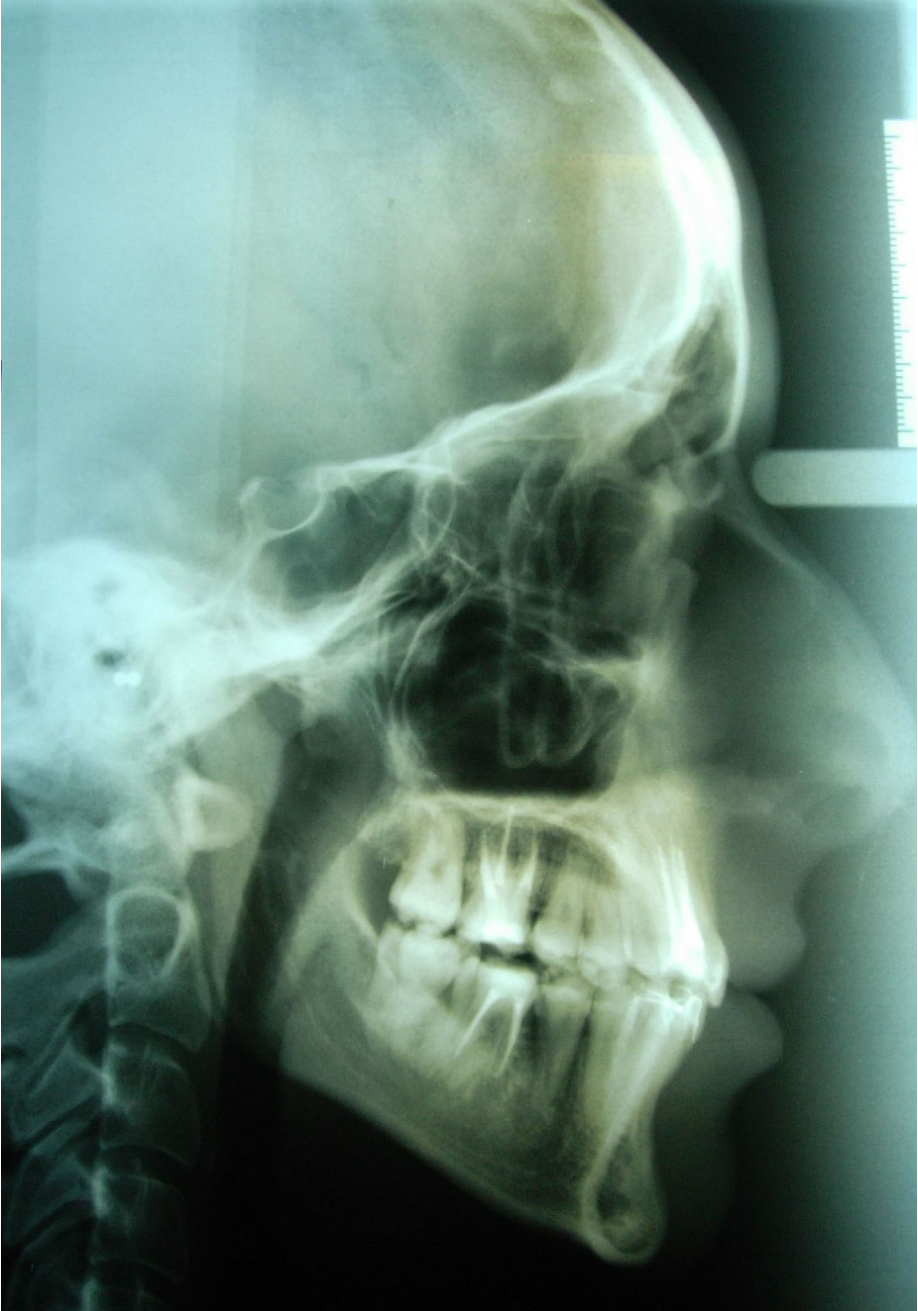
**Final cephalogram of case 1**.

**Figure 13:**
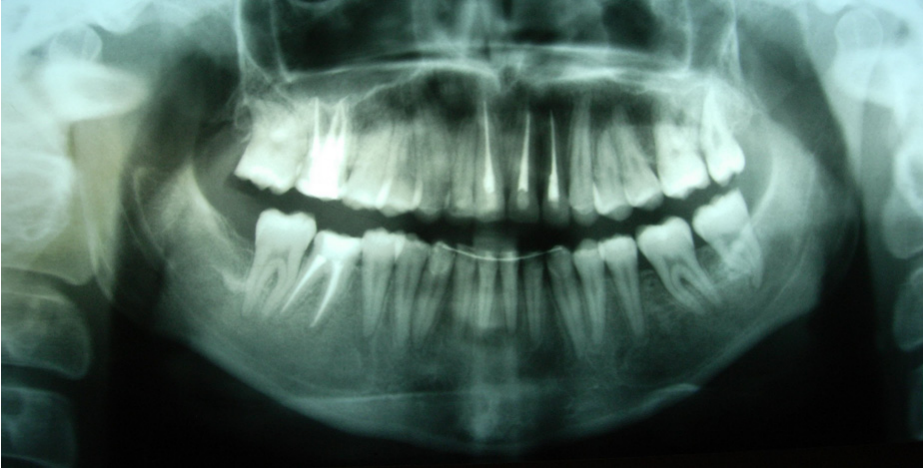
**Final OPG of case 1**.

The treatment was accomplished in 19 months. The crossbite was corrected, and normal overbite and overjet were achieved with Class I canine and molar relationships (Figure 10, 11). The lower left third molar was uprighted. There was a 4-mm increase in the intermolar distance and a 1-mm increase in the intercanine distance. Cephalometric analysis showed insignificant changes (Figure 12, 13 and Table 1).

#### Alternative treatment plans

Expansion of the upper arch could have been performed using quad-helix appliance with or without corticotomy. Limitations of the conventional non-corticotomy expansion include unnecessary expansion on the unaffected side, which might lengthened the treatment time and required special mechanics during the rest of treatment to constrict it. Expansion using quad-helix with corticotomy could have been a more efficient method of expansion except for the expected patient discomfort when used with adult patients.

### Case 2

#### History and Diagnosis

**Figure 14:**
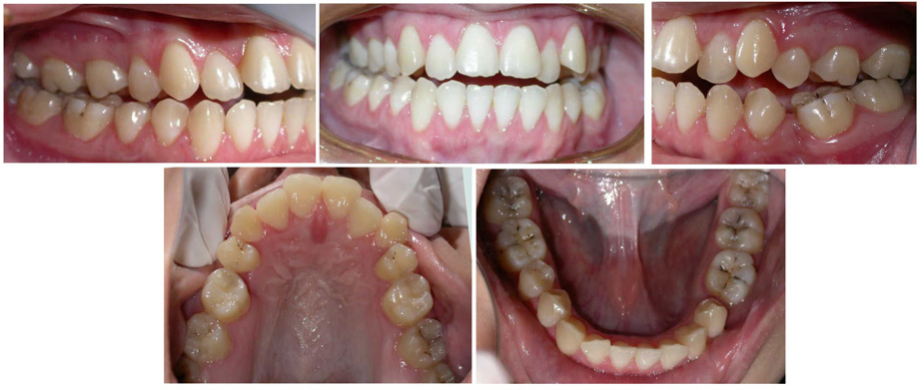
**Initial intra-oral composite photograph of case 2**.

A 24-year-old patient visited the orthodontic clinic with aesthetic concerns related to an anterior open bite. Her medical and dental history was insignificant except for extraction of the upper and lower right second premolars. Bilateral clicking of temporomandibular joint was noticed. The patient was periodontally healthy (Figure 14).

**Figure 15:**
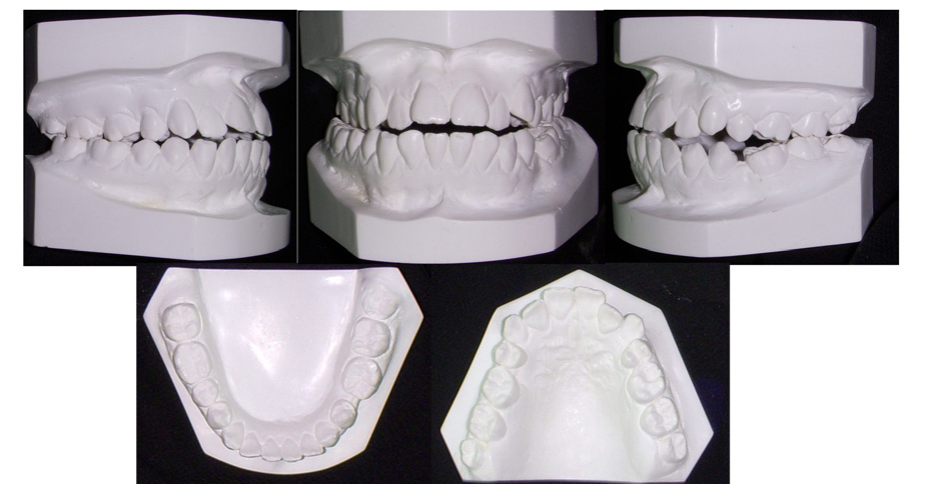
**Initial study model of case 2**.

Clinical examination and the review of records revealed a well-proportioned face with fair symmetry and a normal nasolabial angle. The upper and lower midlines were coincident with the facial midline. The patient had an Angle Class II molar relationship and a Class III canine relationship due to missing premolars. There was a bilateral crossbite that was more severe on the right side than the left side, an anterior open bite of 2-3 mm, an impacted upper left second premolar, and a moderate crowding in the upper and lower arches (Figure 15).

**Figure 16:**
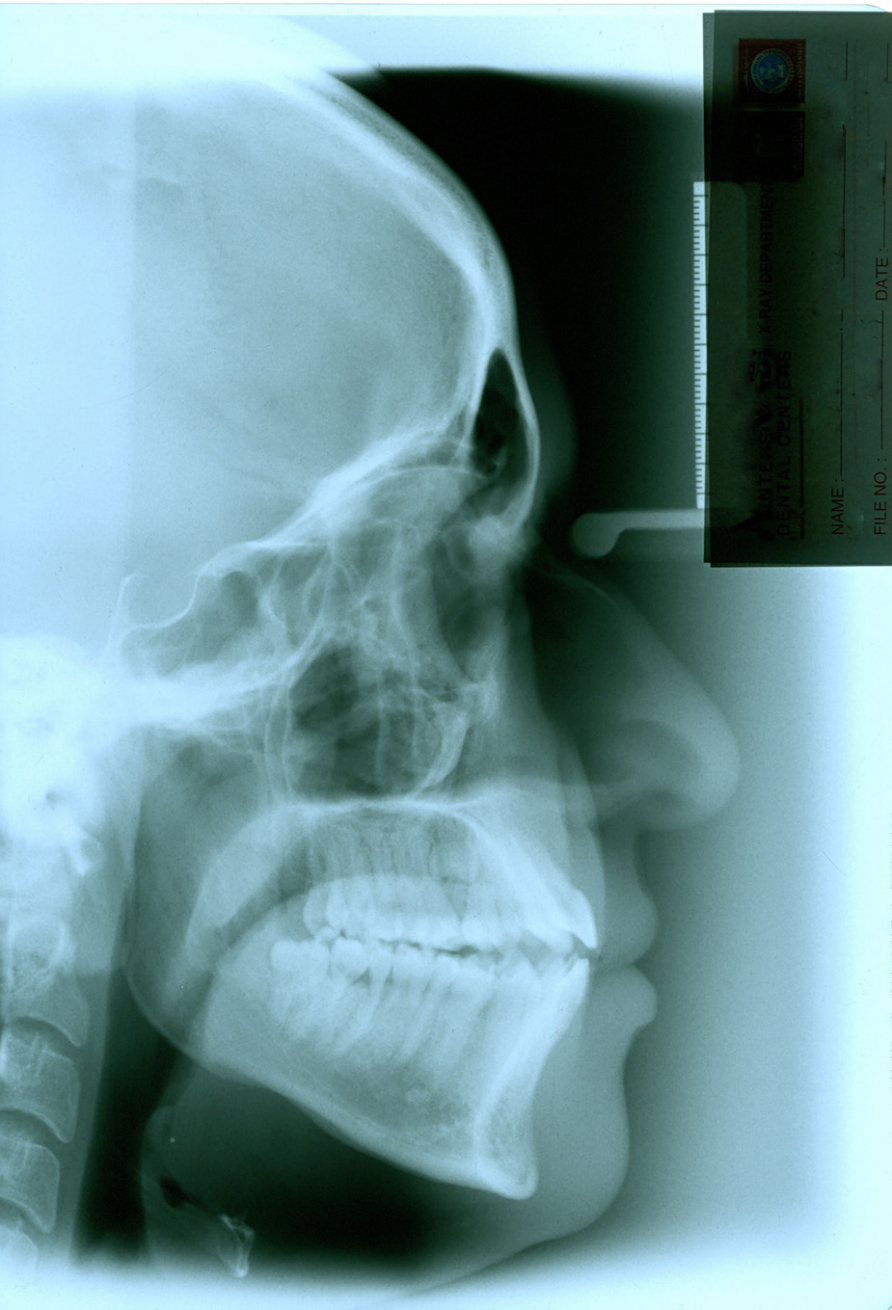
**Initial cephalogram of case 2**.

**Figure 17:**
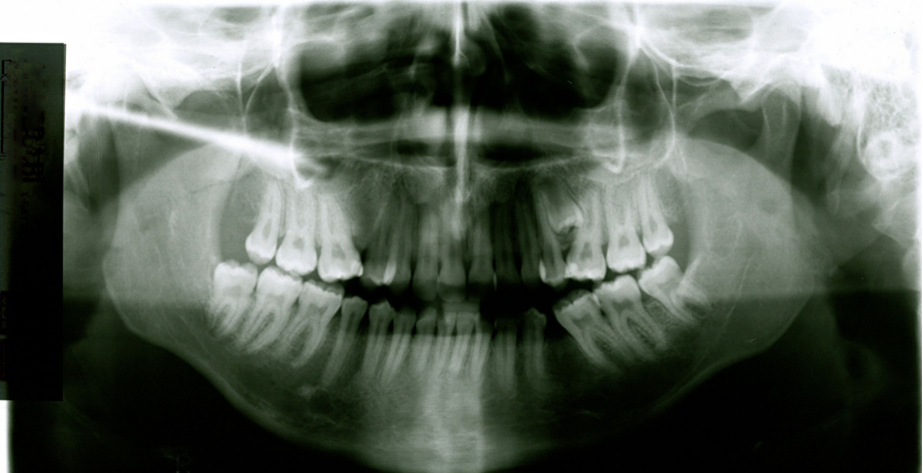
**Initial OPG of case 2**.

Radiographic evaluation revealed a Class I skeletal relationship with a slightly increased mandibular plane angle, proclined upper incisors, and a slight increase in lower facial height (Figure 16, 17 and Table 2). Upper left second premolar was impacted with incomplete root formation.

**Table 2:** Initial and final cephalometric readings of case 2.

Measurements	Norms*	Pre	Post
**Facial angle NPog/SN**	80°°	79°	79°

**SNA**	82°	80°	80°

**SNB**	80°	77°	77°

**ANB**	2°	3°	3°

**NA/APog**	0°	3°	2°

**Mandibular Plane to FH**	25°	30°	31°

**Mandibular Plane to SN**	32°	33°	34°

**Y Axis (SGn/SN)**	60° - 66°	70°	71°

**U Incisor to SN**	103°	95°	96°

**U Incisor To NA angle**	22°	18°	18°

**U Incisor to NA Distance**	4 mm	4 mm	5 mm

**L Incisor to Mandibular Plane**	90°	90°	95°

**L incisor to NB Angle**	25°	21°	24°

**L incisor to NB Distance**	4 mm	3.5	5

**Inter-incisal Angle**	130-132°	127°	132°

**ANS to Gn/N to Gn**	57%	55%	56%

* Steiner CC, Cephalometric for you and me. American journal of orthodontics. 1960; 46: 721

#### Treatment Objectives

- To resolve the posterior crossbite differentially via corticotomy-assisted expansion.

- To facilitate the eruption of the impacted upper left second premolar via corticotomy and extraction of the adjacent first premolar.

- To resolve upper and lower crowding.

- To resolve the anterior crossbite.

- To achieve a Class I molar and canine relationship with normal overjet, normal overbite, and correct midlines.

#### Treatment Planning and Progress

Corticotomy was performed differentially: buccal and palatal on the right side and only buccal on the left side. Expansion started 10 days post-corticotomy and was done using a quad-helix appliance. After 12 weeks overcorrection was achieved, the quad-helix was removed, and upper and lower pre-adjusted fixed appliances (Victory Series™ low profile brackets, 3 M Unitek, St. Paul, Minnesota, USA. (0.018 × 0.025-in)) were used for aligning, leveling, arch coordination, and finishing. For retention, an upper wrap-around retainer and a lower fixed retainer from canine to canine were used.

#### Results

**Figure 18:**
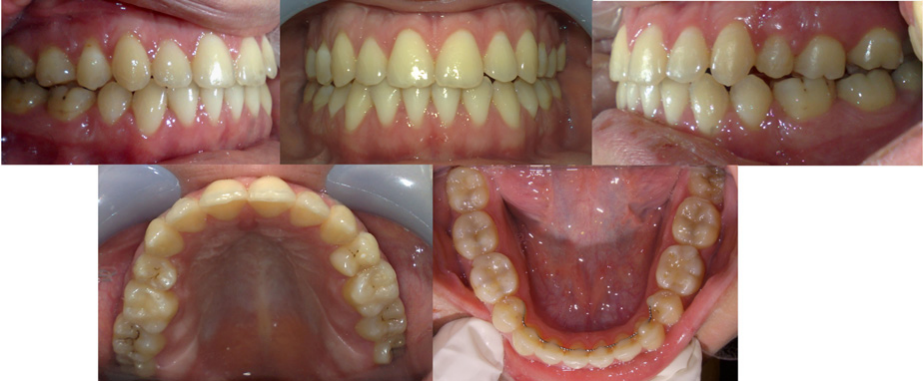
**Final intraoral composite photograph of case 2**.

**Figure 19:**
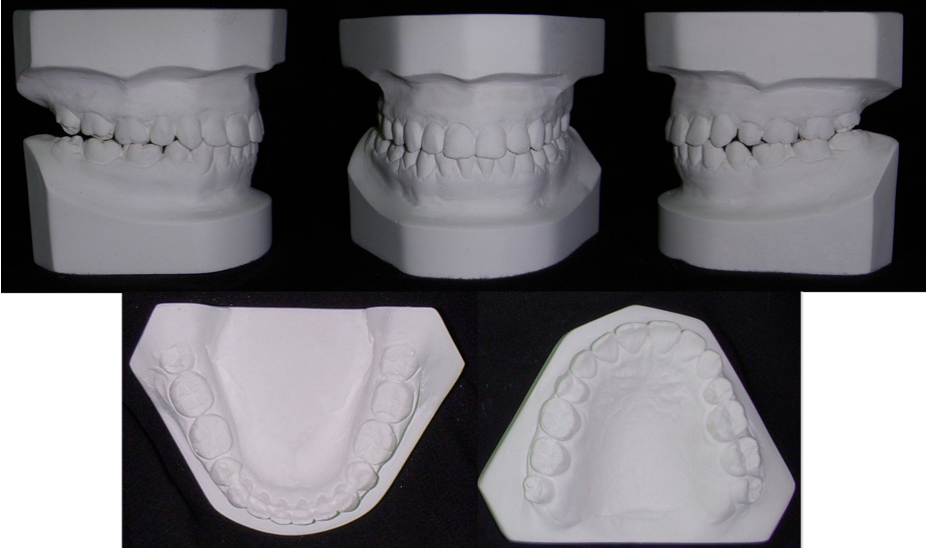
**Final study model of case 2**.

**Figure 20:**
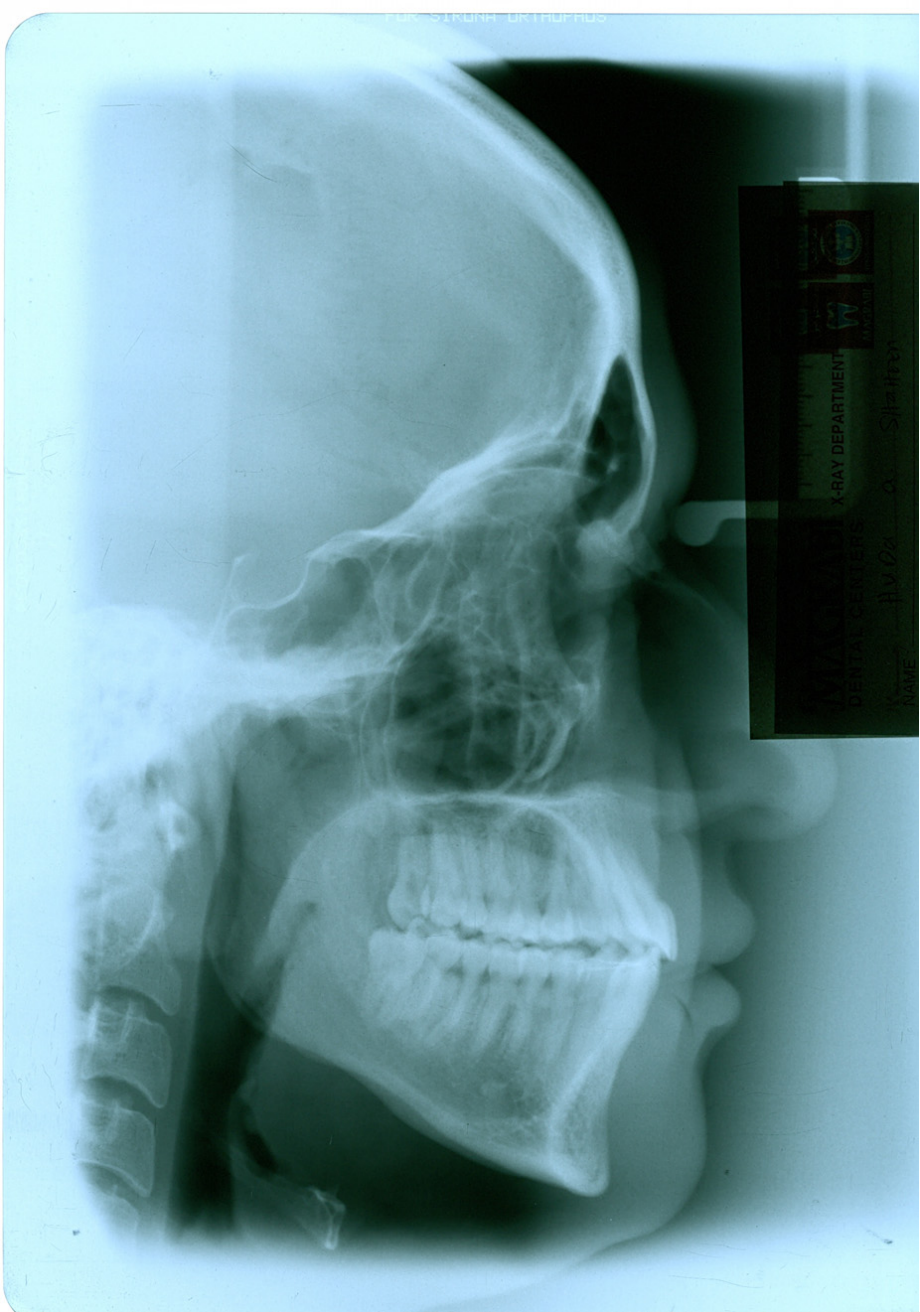
**Final cephalogram of case 2**.

**Figure 21:**
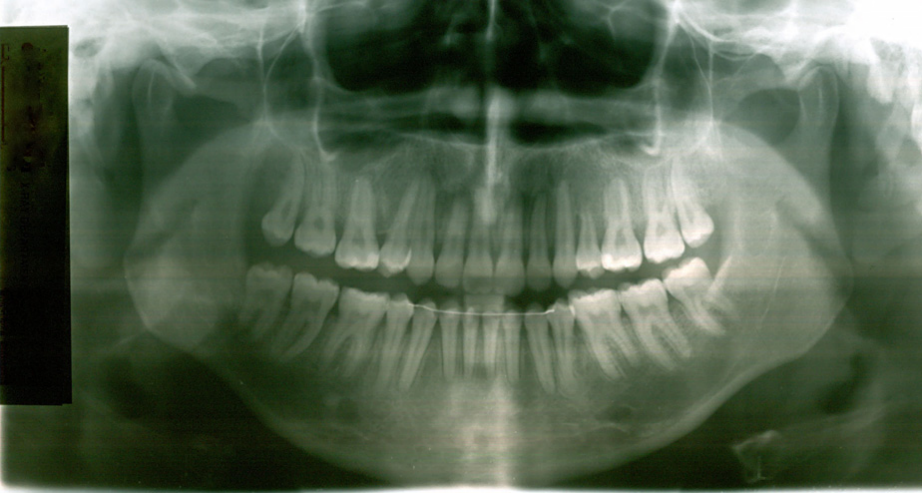
**Final OPG of case 2**.

The treatment duration was 18 months. The crossbite was corrected; normal overbite, normal overjet, and Class I canine and molar relationships were achieved (Figure 18, 19). Intermolar distance and intercanine distance were increased by 3 mm and 1 mm, respectively. Cephalometric analysis showed correction of incisor proclination and maintenance of lower facial height (Figure 20, 21 and Table 2).

#### Alternative treatment plan

Surgically assisted rapid palatal expansion (SARPE) and slow palatal expansion (SPE) using quad-helix appliance could have been alternative treatment options. SARPE could have been a more invasive procedure than CAE, while SPE could have been more risky regarding the periodontal health.

## Discussion

Unilateral transverse maxillary deficiency in adults remains a highly challenging problem to treat; clinicians are usually left with very limited options in these cases. A unilateral effect of expansion is what the clinician desires to accomplish in these cases. Unfortunately, activating palatal expanders always produce a bilateral effect; although several designs and modifications have been suggested, a bilateral effect has always been evident [14-16]. To overcome the unnecessary contralateral expansion in the first case, corticotomy was performed only on the crossbite side to encourage more tissue turnover and accelerate tooth movement on that side, unlike the other side, which experienced regular type of tooth movement. Therefore, expansion occurred faster on the crossbite side than on the normal side. However, some expansion was also observed in the normal side as well, which was mainly due to tipping and relapsed quickly after removal of the expander. On other hand, expansion on the corticotomized side was believed to be bodily in nature and more stable. The relatively shorter duration of cross bite correction, 10 weeks in the first case and 12 weeks in the second one, is considered as an additional advantage of the technique. Another advantage of CAE, that was evident in the first case, was the possibility of using simple expansion appliances, unlike the methods of slow expansion and surgically assisted expansion, which require bulky conventional palatal expanders. Heavy labial wire combined with a regular fixed orthodontic appliance can be adequate for producing the desired results, especially for moderate types of crossbite. This could be ideal for adult patients who do not tolerate palatal expanders. However, palatal expanders such as the quad-helix appliance or the Hyrax-type palatal expander are still considered more efficient options for the more severe forms of constriction. Appliance selection is also important to ensure normal healing after corticotomy. For example, the Hass-type palatal expander should not be used in conjunction with corticotomy to avoid any ischemic effect on the palatal side.

Post-treatment, the inter-molar distance was increased; 3 mm in the first case and 4 mm in the second case, over the initial measurement. However, the argument always remains regarding how much of that expansion was tipping. Using the ruler of the American board of Orthodontics grading system, the level of buccal and palatal cusps of molars and premolars were measured and found to be the same before and after treatment. This indicates that the expansion was bodily in nature, unlike the covenantal methods of expansion in skeletally mature patients where expansion is expected to be tipping in nature [17].

CAE can also be done differentially, according to the severity and side of crossbite, as shown in the second case. Buccal and palatal corticotomy was performed on the more severe side and only buccal corticotomy was performed on the less severe side. This was done to have greater bone turnover and enhanced expansion on the more constricted side than the less constricted one.

Proper treatment planning is required for CAE. The orthodontist should work closely with the periodontist to plan the procedure, the side of the corticotomy, and the teeth that will be involved. Case selection is very critical, as this technique should be limited only to moderate skeletal discrepancies; in no instance should it be a replacement for surgically assisted rapid palatal expansion (SARPE) in severe forms of palatal constriction. A patient with active periodontal disease should be stabilized before any step of CAE is attempted. Periodontal health should be monitored closely by the periodontist during the entire treatment to avoid any periodontal complications such as gingival recession.

The technique of CAE is considered as a relatively invasive procedure when compared to the conventional slow expansion methods, since it requires periodontal surgery. However, invasiveness is considered minimal when compared to the SARPE. Mild and few post-operative complications are expected such as soft tissue edema and mild pain, which can be controlled by non-steroidal anti-inflammatory drugs. Complications such as subcutaneous hematomas of the face and the neck were reported in a single case report following intensive corticotomy [23]. In our reported cases, periodontal health was maintained without any complications.

This report is considered as the first to emphasize CAE as a new indication for PAOO to treat unilateral crossbites and bilateral crossbites with different side severity.

## Conclusions

Unilateral CAE is an effective and reliable technique to treat a true unilateral crossbite. In addition, unilateral buccal and palatal corticotomy on one side and buccal corticotomy on the other side represent an effective method to treat a bilateral crossbite with different side severity in adult patients. CAE offers the use of simple expanders, such as heavy labial wires, combined with regular fixed orthodontic appliances instead of the conventional bulky palatal expanders.

## Competing interests

The authors declare that they have no competing interests.

## Authors' Information

AHH is an associate professor and consultant of orthodontics at King Abdulaziz University (KAU). He has a PhD and certificate of orthodontics from the University of Illinois at Chicago. He is the chairman of the Saudi board in orthodontics- western region of Saudi Arabia (KSA)

ATG is an associate professor and consultant of periodontics at KAU. He is the chairman of Oral Basic and Clinical Sciences Department, and the chairman of Saudi board in periodontics- western region of KSA.

AAF and AAH are senior residents enrolled in the Saudi Board in Orthodontics-Western region of KSA.

## Authors' contributions

AHH performed the orthodontic treatment, analyzed the records, reviewed all patients' data and designed the case report. ATG performed one of the surgical procedures of corticotomy. AAF participated in the orthodontic treatment of the cases, drafted the manuscript and wrote the text. AAH reviewed the manuscript and helped in answering reviewer's comments'. MKH treated the first case under the superiviso of the first author.

All authors read and approved the final manuscript.

## Consent

Written informed consent was obtained from the patients for publication of these case reports and any accompanying images. A copy of the written consent form is available for review by the Editor-in-Chief of this journal.
